# Echocardiographic assessment of atrial, ventricular, and valvular function in patients with atrial fibrillation—an expert proposal by the german working group of cardiovascular ultrasound

**DOI:** 10.1007/s00392-024-02491-6

**Published:** 2024-08-26

**Authors:** Andreas Hagendorff, Stephan Stöbe, Andreas Helfen, Fabian Knebel, Ertunc Altiok, Stephan Beckmann, Tarek Bekfani, Thomas Binder, Aydan Ewers, Ali Hamadanchi, Henrik ten Freyhaus, Thomas Groscheck, Dariush Haghi, Jan Knierim, Sebastian Kruck, Karsten Lenk, Nicolas Merke, Dietrich Pfeiffer, Elena Romero Dorta, Tobias Ruf, Christoph Sinning, Nina C. Wunderlich, Roland Brandt, Sebastian Ewen

**Affiliations:** 1 Department of Cardiology, University Hospital Leipzig AöR, Leipzig, Germany; 2 Department of Kardiologie, Katholische St. Paulus Gesellschaft, St. Marien Hospital Lünen, Lünen, Germany; 3 Department of Internal Medicine II, Cardiology, Sana Klinikum Lichtenberg, Berlin, Germany; 4 Department of Cardiology, Angiology, and Intensive Medicine, University Hospital Aachen, Aachen, Germany; 5 Privatpraxis Kardiologie, Beckmann Ehlers Und Partner, Berlin-Grunewald, Germany; 6 Department of Cardiology and Angiology, University Hospital Magdeburg AöR, Magdeburg, Germany; 7 Department of Cardiology, University Hospital AKH Wien, Vienna, Austria; 8 Department of Cardiology and Angiology, BG University Hospital Bergmannsheil, Bochum, Germany; 9 Department of Cardiology, University of Jena, Jena, Germany; 10 Department of Internal Medicine III, Cardiology, University of Cologne, Cologne, Germany; 11 Kardiologische Praxisklinik Ludwigshafen-Akademische Lehrpraxis of the University of Mannheim, Ludwigshafen, Germany; 12 Department of Internal Medicine and Cardiology, Paulinenkrankenhaus Berlin, Berlin, Germany; 13 Praxis Für Kardiologie Cardio Centrum Ludwigsburg, Ludwigsburg, Germany; 14 Department of Cardiothoracic and Vascular Surgery, Deutsches Herzzentrum Charité Berlin, Berlin, Germany; 15 Kardiologische Praxis Berlin (Adlershof), Berlin, Germany; 16 Department of Cardiology, Angiology and Intensive Care Medicine, Deutsches Herzzentrum Charité Berlin, University of Berlin, Campus Mitte, Berlin, Germany; 17 Department of Cardiology, Center of Cardiology, Heart Valve Center, University Medical Center Mainz, University of Mainz, Mainz, Germany; 18 Department of Cardiology, German Centre of Cardiovascular Research (DZHK), University Heart and Vascular Center Hamburg, Hamburg, Germany; 19 Department of Cardiology, Asklepios Klinik Langen, Langen, Germany; 20 Department of Cardiology, Kerckhoff Klinik GmbH, Bad Nauheim, Germany; 21 Department of Cardiology and Intensive Care Medicine, Schwarzwald-Baar Klinik, Villingen-Schwenningen, Germany; 22 University Heart Center Freiburg • Bad Krozingen, Freiburg, Germany

**Keywords:** Atrial fibrillation, Echocardiography, Left atrial strain, Systolic left ventricular function, Diastolic left ventricular function

## Abstract

Echocardiography in patients with atrial fibrillation is challenging due to the varying heart rate. Thus, the topic of this expert proposal focuses on an obvious gap in the current recommendations about diagnosis and treatment of atrial fibrillation (AF)—the peculiarities and difficulties of echocardiographic imaging. The assessment of systolic and diastolic function—especially in combination with valvular heart diseases—by echocardiography can basically be done by averaging the results of echocardiographic measurements of the respective parameters or by the index beat approach, which uses a representative cardiac cycle for measurement. Therefore, a distinction must be made between the functionally relevant status, which is characterized by the averaging method, and the best possible hemodynamic status, which is achieved with the most optimal left ventricular (LV) filling according to the index beat method with longer previous RR intervals. This proposal focuses on left atrial and left ventricular function and deliberately excludes problems of echocardiography when assessing left atrial appendage in terms of its complexity. Echocardiography of the left atrial appendage is therefore reserved for its own expert proposal.

**Supplementary Information:**

The online version contains supplementary material available at 10.1007/s00392-024-02491-6.

## Introduction

In contrast to the catalog of the “International Classification of Diseases” (ICD-10: I48) and the frequently published opinion of the majority of rhythmologists, atrial fibrillation (AF) is not to be understood as a clearly defined disease (ICD-10), but rather as a symptom or syndrome such as angina pectoris, dyspnea, heart failure (HF) or fever. Often AF is part of a syndrome with several additional symptoms, regardless of whether we can distinguish the natural history and consequences of AF on morphology and hemodynamics.

AF may have a genetic [1], morphologic [2], hemodynamic [3], structural [4, 5], inflammatory [6], ischemic [7, 8], electrophysiologic [9], autonomic nervous disorder, and/or toxic history that contribute to the occurrence, persistence, and risk of arrhythmia (Table 1).

**Table 1:** Causes of atrial fibrillation and traceability by ultrasound

Natural history of AF	Detectable using TTE/TEE	Non-detectable using TTE/TEE
Genetic [1, 2]		◾ Congenital Genes for AF
Morphologic [3, 4]	◾ Dilatation of chambers	
	◾ Ventricular hypertrophy	
	◾ Intracardiac masses	
	◾ Shunts	
	◾ Degenerative scar in atrial wall	
	◾ Congenital heart disease	
Hemodynamic [5, 10]	◾ Systolic/diastolic heart failure	
	◾ Dynamic valvular regurgitation	
Structural [13]	◾ Congenital heart disease	
	◾ Postsurgical scar	
Inflammatory [6]	◾ General/diffuse hypokinesia	
	◾ Markers of myo- or endocarditis	
	◾ Pericarditis, effusion	
	◾ Dressler’s syndrome	
Ischemic/postischemic [7, 8]	◾ Regional hypokinesia	
	◾ Postischemic scar, aneurysm	
Electrophysiologic [9]		◾ Atrial/atrioventricular tachyarrhythmias
		◾ Pulmonary vein foci Autonomic imbalance
		◾ Preexcitation syndrome
		◾ Pacing-induced
		◾ Brady/tachy-syndrome
Post cardiac surgery [12]	◾ Hemodynamic consequences of prosthetic valves	
	◾ Scar after cannulation of IVC/SVC for extracorporeal circulation	
	◾ Scar after atriotomy or vents	
	◾ pericardial constriction	
Toxic		◾ e.g., alcohol and drug abuse

*AF* atrial fibrillation, *TTE* transthoracic echocardiography, *TEE* transesophageal echocardiography

In most AF patients, the first step after electrocardiographic diagnosis is a morphological and hemodynamic cardiac examination using echocardiography and Doppler echocardiography. If significant valve dysfunction, hypertrophy, intracardiac tumors or shunts are detected, the origin of AF is obvious. If global or regional wall motion abnormalities with or without HF [5, 10] are detected, the question arises whether diffuse myocardial disease such as myocarditis, 3-vessel coronary artery disease or cardiomyopathy could be the cause of AF or whether tachyarrhythmia is followed by arrhythmogenic cardiomyopathy in the long term [11]. If no pathologic or hemodynamic wall motion abnormalities can be documented, the question arises whether minimal changes are not detected—which should be verified by echocardiography follow-ups—or whether a genetic, electrical, or toxic natural history is responsible for AF occurrence (Table 2). The diagnostic strategy should include also the family history of AF or known cardiac arrhythmias. AF after cardiac surgery depends on several factors like local inflammation, electrolyte imbalances, sympathetic activation, and scar formations [12]. The following text offers recommendations for comprehensive evaluation of AF patients using echocardiography.

**Table 2:** Observations in AF patients by echocardiography and their consequences

Observation	Explanation of AF	Consequence for treatment
◾ Moderate/significant valve dysfunctions	◾ Probable cause of AF onset	◾ Treatment of morphologic/hemodynamic abnormalities followed by treatment of AF later
◾ Shunts		
◾ Intra/pericardiac tumors		
◾ LV/RV hypertrophy		
◾ HFpEF		
◾ Pericarditis, Dressler’s syndrome		
◾ Cor pulmonale		
◾ Regional hypokinesia	◾ Additional diagnostics are needed	◾ Treatment of different causes
◾ Global hypokinesia		
◾ ± HFrEF/HFmrEF	◾ Reason or consequence of AF (arrhythmogenic cardiomyopathy)?	◾ Treatment of AF and echocardiographic follow-ups
◾ No morphologic/hemodynamic abnormalities	◾ Minimal changes or genetic/electrophysiologic/ toxic reasons for AF?	◾ Additional diagnostics

*HFpEF* heart failure with preserved ejection fraction, *HFmrEF* heart failure with mildly reduced ejection fraction, *HFrEF* heart failure with reduced ejection fraction, *AF* atrial fibrillation, *LV* left ventricle, *RV* right ventricle

## Diagnostic targets of echocardiography in AF patients

AF is the most common sustained cardiac arrhythmia in adults. The lifetime risk of AF depends on age, and is influenced by genetic and (sub)clinical factors [13]. AF is common in heart failure (HF) with a bidirectional relationship whereby one can precipitate the other. The increased risk of AF patients to develop HF and vice versa is attributable to two factors. Firstly, both entities share a risk profile with several coinciding cardiovascular risk factors, increasing the odds of developing both conditions separately from each other. Secondly, they share pathophysiological pathways, and as such can provoke and sustain each other [14, 15]. A comparison within HF spectrum indicated that AF was progressively more common in HF patients with preserved left ventricular (LV) ejection fraction (EF) than with mid-range or reduced LVEF [16]. The 2020 ESC Guidelines on AF recommend transthoracic echocardiography (TTE) as part of the first line and most widely used imaging technique in all AF patients to guide management [13]. The echocardiographic challenge in AF patients concerns the detection of the AF consequences on the size of cardiac cavities and their functional alterations as well as consecutive valvular functions—especially in the case of valvular defects. In general, echocardiographic analysis in AF patients is important for characterizing individual hemodynamics and prognosis.

Arrhythmogenic cardiomyopathy is a potentially reversible variant of non-ischemic cardiomyopathy induced or mediated by both, supraventricular and ventricular rhythm disorders, with AF being the most common cause. The degree of LV systolic dysfunction correlates with the duration and rate of the tachycardia leading to cellular and extracellular changes [17]. Uncontrolled LV excitations and LV contractions during AF can also precipitate functional mitral regurgitation (MR) and cause a rate-related left bundle branch block (LBBB), both with a negative impact on cardiac output (CO). Given the high prevalence of AF in HF patients, a common clinical problem is to determine whether the tachycardia is the initiator of the cardiomyopathy or consequence of an unknown cardiomyopathy of different etiology. The hallmark of this condition is partial or complete reversibility within a few weeks to months following rhythm and rate control. Myocardial deformation imaging has the potential to predict functional recovery in presumptive tachycardia-induced cardiomyopathy based on the longitudinal strain pattern. Relatively preserved apical strain, a pattern also seen in cardiac amyloidosis, was found in patients without improvement in LVEF, whereas a greater reduction in apical strain correlated with improvement in LVEF [18]. AF patients develop a mild to moderate decline in LV performance with a return to the previous baseline following restoration of SR. The increase in LV stroke volume (LVSV) and LVEF immediately after cardioversion in the setting of an unchanged intrinsic cardiac contractility are most likely due to enhanced LV diastolic filling resulting from an increase in cycle length in combination with a return of LA contractile function [19].

## General problems using echocardiography in AF patients: “mean values,” “maximum values,” or “index values”—what is recommended? What is useful?

Echocardiography in AF patients can be challenging due to higher heart rates and beat-to-beat variability, respectively. Therefore, it is mandatory to consider heart rate and the amount of arrhythmic burden with their influence on functional parameters. The joint guidelines on cardiac chamber quantification suggest to average the respective echocardiographic parameters over at least five beats in AF to account for inter-beat variability, but this recommendation is only based on consensus opinion [20]. Alternatively, the index-beat assessment by choosing appropriate cardiac cycles after nearly equal preceding and pre-preceding RR intervals with similar RR intervals and preferably an equivalent heart rate less than 100 bpm is a reliable and reproducible approach [21–25]. With increasing heart rate, diastole is reduced. Therefore, the cycle length of the preceding RR interval determines LVSV [21, 26, 27]. The relationship between the increase in heart rate and the decrease in systolic function in AF can be explained by the force-frequency relationship of the cardiac muscles when loading conditions change [28]. In AF patients the variability of systolic function is reduced, if the preceding RR intervals are quite long or similar in length as in bradyarrhythmia [21, 28].

A pronounced variability of systolic function—and also in leaflet and cusp mobility—can be observed with strongly varying heart rates, especially in brady-tachycardia syndrome [27, 29, 30]. Measurements using the index beat method seem to be recommended—even for strain analyzes—only for AF patients whose cardiac cycle intervals do not differ by more than 60 ms [31]. Lower variations of parameters measured by Doppler echocardiography were observed with increasing LV filling pressures [32].

In AF patients, a distinction must be made between the functionally relevant status, which is characterized by the averaging of the measurement results of hemodynamic parameters, and the best possible hemodynamic status, which is achieved with the most optimal LV filling according to the index beat method with longer previous RR intervals.

Due to the varying RR intervals in AF patients, the results of volumetric and functional measurements vary with different chamber filling properties. Thus, echocardiographic measurements are best performed in AF patients after rate control [33–35]. To increase the reproducibility of volumetric and functional measurements, the "short—long—long" approach should be used in AF patients. It should be aimed to perform measurements within similar RR intervals or, if possible, to perform measurements after 2 longer RR intervals. However, it must always be considered that the measurements with longer RR intervals represent the state of best possible contractility with best possible LV filling, but not the average functional state with rapidly changing heart rates. The evaluation of CO within multiple heart cycles by three-dimensional (3D) echocardiography had a higher accuracy compared to a single measurement in AF patients regardless the heart rate [36]. Further, 3D echocardiography can overcome foreshortening. In addition, 3D and multiplanar echocardiography allow to perform measurements, as for example LV volumes and LV ejection fraction, at the same cardiac cycle on different planes, a clear benefit in patients in AF.

The following practical approach can be recommended in AF patients. Comparable time intervals of cardiac cycles should be preferably evaluated, if possible after rate control. If different RR intervals are present, the average cardiac function is determined by averaging results of 5–10 heart beats.

**Fig. 1:**
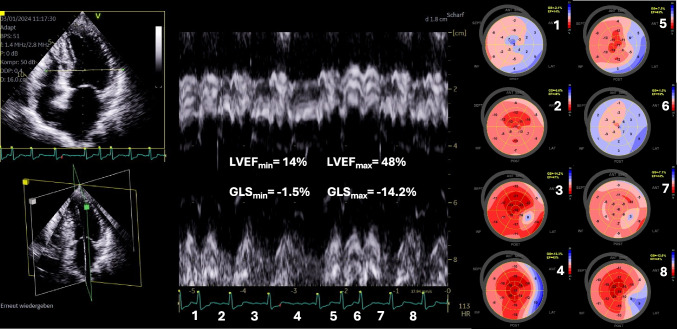
Beat-to-beat variability of left ventricular (LV) deformation in a patient with tachyarrhythmia in the range of 115 bpm. The anatomical M-Mode of a basal 4-chamber view is shown left sided (septum above—lateral wall below) illustrating the different beat-to-beat contraction amplitudes reflected by radial wall motion. The minimum and maximum values for LV ejection fraction (LVEF) and global longitudinal strain (GLS) are presented in the M-Mode. The numbers represent the consecutive cardiac cycles. For each cardiac cycle, the respective LV longitudinal deformation pattern (bull’s eye) is shown labeled by the respective number right sided. The shortest cardiac cycle after a preceding short cycle showed the lowest LV deformation (cycle 6). The longest cardiac cycle after a preceding normal cycle showed highest LV deformation (cycle 3). The minimum and maximum values for LVEF and GLS are presented in the M-Mode. This example illustrates the correlation of global LV deformation with cycle length

The impact of AF on radial contraction and on LVEF and global longitudinal strain (GLS) is presented in Fig. 1 and in the Supplement Material (Supplementary Figure S1–S3).

## Analysis of LV systolic function in AF patients

AF is frequently associated with LV systolic dysfunction or its exacerbation by worsening hemodynamics and tachycardia-induced cardiomyopathy in up to 50% of the patients [22, 37]. The lack of atrial systole determines a lower preload, the RR interval variations modify LV filling and LV output according to the Frank-Starling mechanism and affects LV contractility by the pressure-interval or force-interval relationship, and tachyarrhythmia reduces LV filling time [24, 35]. The beat-to-beat variation, which is characteristic for AF, can be a detrimental factor to the reliable and reproducible clinical assessment of LV systolic function [38]. Automatic echocardiographic approaches to improve the assessment of LV function in AF are being used more and more frequently in clinical routine, although many investigators still rely only on eyeballing.

LVEF, mitral annular plane systolic excursion (MAPSE), *s*′, and global longitudinal strain (GLS) are also used in AF patients to characterize LV systolic functions. All parameters show significant variabilities at different heart rates. LVEF is still the most used parameter in clinical practice to evaluate LV systolic function. However, echocardiographic assessment of LV longitudinal function may provide additional and/or incremental prognostic information. MAPSE using guided 2D M-Mode imaging is a direct, early, and sensitive index of LV systolic function, even in the presence of preserved LVEF [39]. The measurement of s′ at the junction between basal myocardium and mitral annulus by pulsed wave (pw) tissue Doppler is an early marker of subtle LV systolic dysfunction, simple to measure with good reproducibility and feasibility. The pw tissue Doppler parameter *s*′ was also shown to correlate strongly with the ratio of pressure change in LV cavity during isovolumetric contraction period (dP/dt) [40]. Speckle tracking echocardiography is an optimal tool to quantify accurately LV longitudinal function by GLS in AF patients due to simultaneous triplane or 3D image acquisition. GLS has recently been shown to be associated with embolic stroke among AF patients [41]. For the risk prediction of cardiovascular events, a GLS cut-off of − 12.5% has been proposed [42]. In AF patients who had suffered a myocardial infarction, lower LVEF was associated with an increased risk of 30-day mortality [43], but did not appear to predict long-term mortality in AF patients.

## Analysis of LV filling in AF patients

The main mechanism to impaired systolic and diastolic LV function is the reduced ability of LV relaxation and the increased LV filling pressure in diastole [44]. The diastolic parameters maximum transmitral blood flow velocity (*V*_max_*E*), maximum systolic basal myocardial velocity (*V*_max_*E*´), E-deceleration time (E-DT), and the *E*/*E*′-ratio are the most common parameters. They have also proven to be very robust, valid and highly reproducible in patients with non-valvular AF [10], although evidence is mostly derived from single-center observational studies [32, 45–48]. *V*_max_*E* represents LV early filling and depends on LA to LV pressure gradient. It is highly pre- and afterload dependent [44],

Other Doppler parameters should be considered in AF patients to estimate LV diastolic function and LV end diastolic pressure (LVEDP) with different cut off values in comparison to patients with SR: the peak acceleration rate of mitral E velocity (pathologic > 1900 cm/sec^2^), the isovolumetric relaxation time (IVRT) (pathologic < 65 ms), E-DT (pathologic < 160 ms), deceleration time of pulmonary venous diastolic velocity (pathologic < 220 ms), and E/mitral Vp (*E*/Vp) (pathologic > 1.4), whereas Vp is transmitral flow propagation velocity [49]. The absolute *V*_max_*E* should not be used exclusively for diagnosis of diastolic dysfunction (DD), although a *V*_max_*E* > 200 cm/s indicates increased filling pressure. In AF patients, smaller variations of *V*_max_*E* with a coefficient of variation of 6 cm/s + 2 [32] indicate higher LV filling pressure [50].

Early diastolic velocity *V*_max_*E*′ reflects LV stiffness resulting from active LV relaxation, restoring forces and LV compliance in SR. However, a statement about LV stiffness is almost impossible with varying RR intervals. In addition, LV stiffness is influenced by LV properties like wall thickness, possible fibrosis, scars, inflammation, and cardiac storage diseases. Age-specific normal ranges apply, as compliance slowly decreases with age. *V*_max_*E*′ is the most feasible, reproducible and prognostic most relevant of these Doppler parameters [49]. However, no special cut-off values of *V*_max_*E*′ for AF patients are given. *V*_max_*E*′ is less load dependent than *V*_max_*E* although increased preload—and therefore higher LA-LV gradients—leads to increased *V*_max_*E*′ when LV relaxation remains in normal ranges [51]. In AF patients, afterload is often reduced due to high heart rate and reduced LVSV and, LV relaxation (and *V*_max_*E*′) is reduced and prolonged with respect to previous cycle length and consecutive LV filling. For *V*_max_*E*′ either septal *V*_max_*E*′, lateral *V*_max_*E*′ or an average of both can be analyzed. In contrast, time delay between onset of *E* and *E*′ (measured from ECG R-wave) correlates with impaired LV relaxation independent of mitral valve diseases [52].

As *V*_max_*E*′ corrects *V*_max_*E* for LV relaxation properties, the ratio *E*/*E*’ correlates to pulmonary capillary wedge pressure (PCWP) and therefore LA filling pressures in AF patients [53] as well as in SR [46, 48]. *E*/*E*′ is especially valid in patients with preserved LVEF but not in conditions with reduced LVEF significantly influencing *V*_max_*E* or *V*_max_*E*′. Filling pressures can be assumed normal if *E*/*E*′ is < 8 and being increased when exceeding. In AF patients cutoff value for septal* E*/*E*′ is 11 [49].

E-DT reflects the rate of decrease rate of LA-LV pressure gradient during early LV filling and depends on LV relaxation and restoring forces. In patients with reduced LVEF, a E-DT < 160 ms indicates a restrictive filling pattern with elevated LA as well as LV pressures which is common in AF patients at early stages. Nevertheless, increased LV filling pressures may be present with prolonged E-DT but still normal LA pressure. Restrictive filling is of an overall unfavorable prognosis in AF patients with symptomatic chronic HF irrespective of LVEF [54].

Standardized and proper documentation is necessary for verifiable and reliable assessment of all Doppler parameters considering all aspects of echocardiographic image acquisition [20, 49, 55]. Averaging the results of diastolic echocardiographic parameters over 5–10 consecutive cardiac cycles or using the index beat approach in AF patients depends on the objective, the assessment of the functionally relevant or hemodynamically best possible status. Diastolic function in symptomatic AF patients with normal or preserved LVEF can be characterized by stress testing during physical exertion by ergometry, squats, or handgrips. However, all these tests are limited by the variability of heart rate.

## Analysis of left atrial (LA) morphology and function in AF patients—discussion of corresponding parameters for right atrial (RA) morphology and function

Characterizing of LA morphology and function by echocardiography and other imaging modalities was shown to be essential—especially to improve the detection of elevated LV filling pressures in AF patients with different cardiovascular diseases [56–59]. LA dilatation is associated with adverse cardiovascular outcome [60] and typically occurs secondary to increased LA pressures that augment LA wall tension. Furthermore, LA dimensions directly correlate with the incidence of AF [61], and help to predict AF onset and/or recurrence [62–64]. In the context of AF, the concept of an “atrial cardiomyopathy” was introduced to account for the structural, contractile and electrical adaptations of the atria [65]. LA fibrosis occurs as a consequence of AF but may also be a causative factor, and the degree of fibrosis is a predictor of AF recurrence [66] and promotes LA dilatation [67]. Therefore, detailed characterization of LA morphology and function is important in AF.

LA size in AF patients is obviously variable with different LA filling in relation to the heart rate. The antero-posterior LA diameter, easily and traditionally measured in the parasternal long-axis view at end-systole, is the most extensively studied parameter of LA size in AF. The upper limit of the normal value is set at 40 mm [20]. However, the LA enlargement during LA remodeling in AF is not symmetrical. The apico-basal diameter (measured in the apical 4- or 2-chamber view) increases prior to the antero-posterior diameter (measured in the left parasternal view) being therefore less sensitive [20].

Thus, LA size assessment by planimetry in the 4- and 2-chamber may overcome these limitations. The upper normal limit of the LA area is 20 cm^2^ [20]. However, as compared to LA volume assessment, this parameter is less reliable and is associated with substantial misclassification of LA size even when the LA antero-posterior diameter is added [68]. LA volume better reflects the asymmetric remodeling of the LA. In 2D echocardiography, the biplane (4- and 2-chamber view) modified Simpson’s method of disks is the preferred method to assess LA volume according to current guidelines [20]. If only a single-plane assessment in the four-chamber view is applied, LA volume is typically slightly smaller than in biplane measurements (by 1–2 ml/m^2^) [69]. From a technical point of view, it is mandatory to avoid LA foreshortening and to exclude both, left atrial appendage (LAA) and pulmonary veins, while tracing the endocardial border. LA volume depends on gender and body size. Indexing for body surface area is mandatory to account for these differences. Although it is known that LA volume increases with age and has some variation depending on ethnicity [70], the upper normal indexed maximal LA volume (LAVI_max_, measured immediately before mitral valve opening) was set to 34 ml/m^2^ in all subgroups [20]. For 3D echocardiography, no normal values exist in the guidelines. However, the 95% confidence interval of LAVI_max_ was 23.1–27.3 ml/m^2^ in a recent large meta-analysis including 15 studies and 4226 patients [70]. Of note, this study detected significant vendor-dependent differences in 3D LA volumes. With respect to other volumetric measures, some studies indicate that LA minimal volume (LAVI_min_, measured at mitral valve closure) may closely relate to occurrence of AF episodes in paroxysmal AF [71] and predict elevated LV filling pressures [72]. However, reproducibility of LAVI_min_ appears to be lower and interobserver variability higher compared to LAVI_max_ [73]. LA emptying fraction calculated as LA maximal volume-LA minimal volume divided by LA maximal volume weakly correlates with AF occurrence and does not provide substantial benefit compared to LA volume [74]. Overall, LAVI_max_ is the current gold standard for the assessment of LA size in AF. Hence, LA volume as a morpho-physiologic biomarker assessed by biplane 2-dimensional (2D) echocardiography has stronger associations with cardiovascular outcomes than by unidimensional measurements [75, 76]. 3D echocardiography can circumvent the limitations of LA volume assessment by 2D echocardiography.

LA enlargement and LA fibrosis are clearly associated with cardiovascular events, particularly with AF [77]. 3D echocardiography enables to assess changes of LA volumes throughout the cardiac cycle [78]. Data given by the current state of the literature indicate that among LA volume parameters, the LAVI_min_ might be superior for risk stratification of cardiovascular outcomes [79]. In contrast to LAVI_max_, LAVI_min_ and the LA reservoir function showed better correlation to LV DD and can be used to predict incident AF [80].

According to current guidelines, the recommended parameter for right atrial (RA) size is maximum volume, indexed to body surface area with a normal range of 25 ± 7 ml/m^2^ in men and 21 ± 6 ml/m^2^ in women [20]. In contrast, in patients with pulmonary hypertension (PH), RA area with an upper limit of normal of 18 cm^2^ is the preferred parameter. The reason for this discrepancy is the large wealth of data that exist for RA area as a prognostic discriminator and for risk stratification in PH in contrast to RA volume [81]. RA volume seems to be less or not predictive for AF recurrence [82]. Therefore, RA morphological parameters play only a minor role in the routine assessment AF patients without PH or tricuspid valve disease. RA remodeling is rarely evaluated in AF patients and data regarding its changes and association with AF occurrence and AF recurrence are scarce. There has been growing interest in recent years in understanding the contribution of RA anatomy and function in various cardiovascular conditions [83]. A recent publication of normal values on RA size and function in a large cohort of healthy individuals using 2D and 3D echocardiography offers new possibilities of throwing light to the role of RA remodeling in cardiovascular disease, including in AF patients [84]. The presence of RA fibrosis in AF patients undergoing ablation has been recently published in a study using cardiac magnetic resonance imaging (cMRI) [85]. The role of phasic volumetric RA function in the field of AF is still poorly examined but appeared prognostic relevant for the incidence of de novo AF in a large multi-ethnic cohort study of healthy people using cMRI [86].

LA mechanics often show changes at early stages before LA enlargement occurs [87–89], and therefore play an important role in risk assessment in AF patients, not only in terms of recurrence [90], but also for the occurrence of AF de novo in high-risk patients [89, 91, 92] as well as in the healthy population [93], underlining their role in the early detection of AF. However, there is still a lack of concrete cut-off values for LA function parameters that can be used for therapeutic decisions.

When characterizing LA function in AF patients by echocardiography, several aspects need to be considered. First, the atria lack an effective booster pump function due to reduced intrinsic contractility [87]. Thus, Doppler-derived parameters such as maximum A-wave velocity from pulsed wave Doppler (pw) or A' wave from pw tissue Doppler imaging are not suitable. Second, parameters such as MAPSE, the phasic volumetric method including the calculated LA emptying fraction (LAEmF) or myocardial deformation imaging are limited by the different cardiac cycle lengths [94]. Third, in both AF and SR, the anatomical challenges of LA border delineation may be a shortcoming in functional LA analysis.

LA reservoir function is partly regulated by LV contraction, especially by the systolic movement of the LV base [87] towards the apex, which can be quantified with mitral annular plane systolic excursion (MAPSE). MAPSE is easy to determine with a high temporal resolution and is largely unaffected by poor image quality, unlike the volumetric approach or speckle tracking. MAPSE is suitable in AF and has shown predictive value as a surrogate for LA reservoir function for risk stratification in AF patients [95]. MAPSE assesses the general change in LV size and represents a simple way of approximating LV function [20]. A decline increases risk for AF occurrence. It even predicts outcome after AF ablation [95].

Nevertheless, MAPSE may also be reduced in AF patients and primary LV myocardial disease, such as hypertrophic cardiomyopathy [96]. In these cases, the tricuspid annular plane systolic excursion (TAPSE) can be helpful as additional parameter for assessing the risk of AF independent of numerous confounding factors, including gender or age [96, 97]. The volumetric method for assessing LA function relies on the measurement of LA volumes at three different times of the cardiac cycle using either 2D or 3D TTE. LA maximum volume (LAV_max_), determined at the end of the LV-systole, LA minimum volume (LAV_min_), at the end of the LV-diastole, and pre-A volume (LAV_preA_), at the onset of the P-wave on the electrocardiogram (ECG) [87], which is not applicable in AF patients. Based on these parameters, different calculations can be done to evaluate reservoir, conduit, and contractile function. Worth mentioning in the setting of AF is the evaluation of the LA total LAEmF (total LAEmF (%) = (LAV_max_ − LAV_min_)/LAV_max_ × 100) as an indicator of the reservoir function [94].

In AF patients, a distinction between passive LAEmF (equivalent to conduit function) and active LAEmF (equivalent to contractile function) cannot be made. Total LAEmF was described as a predictive value for SR maintenance after 12 months following electrical cardioversion [98].

In all morphological LA analyzes, the main limitation of echocardiography is that the extent and location of atrial fibrosis [99], and thus the differentiation between cause and consequence of AF, cannot be clarified. This gap can possibly be closed by highly specialized cMRI analyses [100].

## Assessment of LA function by LA strain—how to measure LA strain? What is measured with LA strain?

**Fig. 2:**
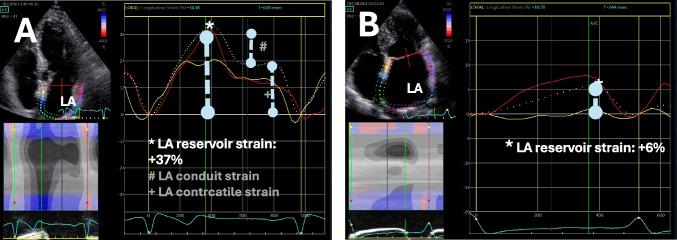
Left atrial (LA) strain analysis in two patients with paroxysmal AF. Patient A with normal LA size and preserved LA reservoir strain. Patient B with dilated LA and a severely impaired LA reservoir strain

Whereas the assessment of LV strain is currently incorporated in most clinical scenarios, the determination of LA strain is not yet used routinely. Increased LV filling pressure, as well as fibrosis and dilatation of the atria, will lead to a reduced LA compliance, which can be characterized by LA strain [101]. LA strain can be derived from all transthoracic apical loops, preferably the apical 4- and 2 chamber views (Fig. 2). LA focused views with a narrow sector without artifacts should be analyzed with respect to optimal image quality. When measuring the LA strain, it appears advantageous to track only the lateral and septal LA wall segments (Fig. 2), since the interatrial septum and the zones of the junctions of the LAA and pulmonary veins do not have any stretchable wall structures or any wall structures at all. However, in contrast to Fig. 2, the LA strain analysis is often evaluated in all LA regions despite this valid methodological limitation. Measuring LA strain is a fast and uncomplicated measurement. LA strain comprises LA reservoir, LA conduit, and LA contraction strain. The most important parameter is LA reservoir strain, as it is the most commonly used parameter and is the only one that can be calculated in the absence of SR [56, 57, 92, 93]. The normal value of LA (reservoir) strain is roughly + 40% [102]. LA strain in relatively observer independent and is not influenced by rhythm state and medical therapies. Therefore is has the potential to be a functional “biomarker” for stroke risk assessment—especially in AF patients [103, 104].

The parameter of LA reservoir strain is included in novel algorithms to assess the risk of elevated LV filling pressures and DD [49]. Recently, LA reservoir strain with a cut-off value of < 18% was added to the ASE/EACVI recommendations to determine elevated LV filling pressures – especially if tricuspid regurgitation is not detectable or other parameters value of the diagnostic algorithm cannot be assessed. Using the LA reservoir strain the accuracy to detect DD could be increased from 68% to a maximum of 80% [105]. Recent data showed that a machine learning based approach could improve risk stratification in grading of DD by including LA reservoir strain [56]. In this study cluster analysis of LA reservoir strain and echocardiographic parameters to grade diastolic function could identify homogeneous groups with distinct diastolic function properties [56]. Regarding the assessed outcome of death and HF related hospitalization this suggested approach was superior in relation the conventional echocardiographic parameters [56].

LA strain is used for risk assessment for LV DD, AF, and stroke, because it offers more information beyond LA size and volumes. LA strain impairment can precede the future development of LV DD [93, 106]. In cryptogenic stroke patients with SR impaired LA strain is a predictor of stroke [107]. Recent studies suggest that LA strain is superior to predict AF compared to LA volumes[108]. LA reservoir, LA contraction and LA conduit strain and the corresponding limits of normality in the healthy participants, were respectively 39.4% (23.0–67.6%), 15.5% (6.4–28.0%), and 23.7% (8.8–44.8%) [109]. In the general population, LA reservoir and LA contraction strain independently predict AF, even in participants with normal-sized LA and normal LV function [93].

Echocardiographic challenges to analyze LA dysfunction during sinus rhythm (SR) in patients with paroxysmal AF are described in the Supplement Material (Supplementary Text).

## Special pathophysiological aspects in AF patients with valvular heart disease—impact of AF on cardiac hemodynamics due to respective valve pathology

The prevalence of AF is increased in patients with valvular heart disease (VHD), particularly in those with pressure or volume overload of the LA. Further, one-third of patients with rheumatic heart disease have AF [110]. Similar findings can be found in patients with tricuspid valve regurgitation (TR), with one-third of AF patients developing at least moderate TR [111]. The incidence of AF is less in aortic valve disease (15%), while AF incidence ranges from 8 to 19% in severe AR [112–114]. Acute valvular heart diseases, such as acute MR due to papillary muscle rupture, can manifest as AF.

**Fig. 3:**
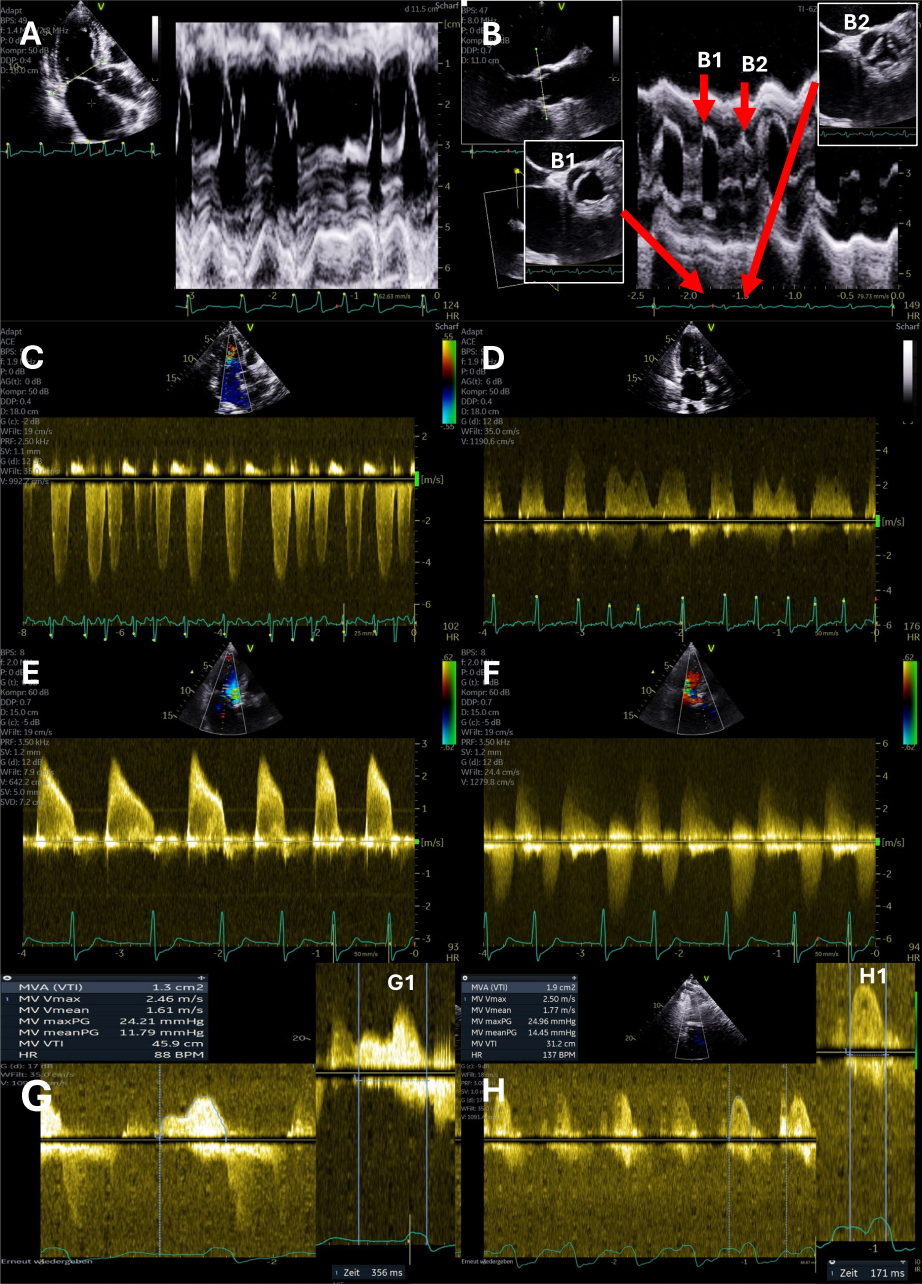
Effects of valvular heart diseases on hemodynamics in patients with atrial fibrillation (AF). **A** Anatomical M-Mode through the mitral valve (MV) separation during tachyarrhythmia—reduced valve separation is documented with shortening of RR-intervals; **B** biplane TTE (transthoracic echocardiography) views and anatomical M-Mode through the aortic valve separation area during AF—reduced valve separation is documented with shortening of RR-intervals—in addition, differences of maximum aortic valve (AV) opening in short axis views at representative RR-cycles (B1, B2); **C** continuous wave (cw) Doppler spectrum of the MV in a patient with mitral regurgitation during AF—reduction of MV inflow and increase of regurgitant flow is documented with increasing heart rate; **D** cw Doppler spectrum of the AV in a patient with aortic regurgitation during AF – increase of AV regurgitant flow is documented with increasing heart rate; **E** cw Doppler spectrum of the MV in a patient with mitral stenosis (MS) documenting the impact of increasing heart rate on transmitral flow; **F** cw Doppler spectrum of the AV in a patient with aortic stenosis documenting the impact of increasing heart rate on transaortic flow; **G** cw Doppler spectrum of the MV in a patient with MS (transmitral velocities and gradients are shown as well as diastolic duration in G1); **H** cw Doppler spectrum of the MV in the same patient with MS after switching to tachyarrhythmia (transmitral velocities and gradients are shown as well as diastolic duration in G2 documenting the impact of AF on transmitral inflow and transmitral gradient)

AF adversely affects the hemodynamics of VHD by eliminating atrial contraction (atrial kick) and shortening diastolic filling periods, reducing CO, and increasing LV filling pressures. In addition, atrial contraction is particularly important in AS patients, as patients commonly have stiff ventricles and rely even more on atrial contraction for LV filling. AF negatively impacts the prognosis of AS [115]**.** The impact of AF on the hemodynamics and prognosis in AR is less studied, but loss of atrial function increases LV filling pressures and exacerbates symptoms. Similarly, AF affects patients with MR, worsening cardiac function and increasing filling and pulmonary pressures. Severe MR complicated by AF is associated with excess mortality [116]. AF can significantly contribute to the clinical worsening and mark a turning point in the natural history of VHD, potentially triggering valve intervention [117]. In cardiac surgery, AF significantly worsens postoperative hemodynamic function and increases the risk of complications [118]. Preoperative AF episodes are also likely to increase the risk of postoperative AF in cardiovascular surgery [119]. AF can not only be caused by VHD but can also worsen or trigger MR and TR. The role of AF in regurgitations is exemplified by the fact that AF patients frequently show improvement in the severity of valvular regurgitation after restoration of SR. Chronic AF contributes to LA enlargement and atrial functional MR. This forms a vicious cycle where AF and MR are involved in the progression of regurgitation. The assessment of VHD severity is challenging in AF patients. AF causes beat-to-beat variations of hemodynamic variables such as stroke volume, regurgitant volume, atrial- and pulmonary pressures affecting measurements used for quantification of VHD, e.g., maximal and mean velocities and gradients, regurgitant volumes, and pressure-half time (PHT) (Fig. 3). In addition, the quantification of LV systolic function, which is important for treatment decisions, is more difficult in AF patients. As mentioned above, it is recommended to average measurements of several heartbeats, choose R-R intervals close to normal heart rates, avoid short diastoles, and document heart rates at which gradients were assessed. In summary, the close association between VHD and AF should be considered when interpreting and managing these conditions.

### “How to do’s” in echocardiography of AF patients with valvular heart disease

AF and AS coexist in patients increasing with age. Up to 50% of patients undergoing transcatheter aortic valve replacement (TAVR) have AF [120]. In addition, AS patients with AF have a worse prognosis compared to patients in SR [115]. In patients with tachyarrhythmia or severe bradycardia the degree of AS should not be assessed only by transvalvular peak velocity and mean gradient (PG_mean_) due to their flow dependence on stroke volume. It is favorable to measure aortic gradients within similar R-R intervals at normal heart rates rather than at extremely short or long intervals, although this will underestimate the functional degree of AS on average. Further, it is recommended to perform measurements in compensated volume conditions. The classification of AS severity remains challenging due to the variable cardiac cycles and the consecutive different LV stroke volumes in AF patients, leading to different beat-to-beat effective valve orifice area (EOA) calculated by the continuity equation. Surprisingly, current recommendations do not indicate information on methodological aspects of echocardiographic quantification of AS severity in patients with AF [121]. Whereas in scientific studies up to 15 RR intervals are recommended to be averaged [122], it is recommended to average measurements of mean gradients (PG_mean_) of 3 to 10 consecutive beats by current guidelines, because the highest velocity could overestimate and the lowest velocity could underestimate AS severity [123]. This is of particular importance if the PG_mean_ and the valve orifice area are discordant to define AS severity. To overcome limitations due to different cycle length, the dimensionless velocity index (DVI) can be used, whereas DVI is the ratio of the velocity–time-integral (VTI) determined in the LV outflow tract (LVOT) and transvalvular through the aortic valve (VTI_LVOT_/VTI_AV_). DVI < 0.25 is highly suggestive for severe AS [121]. In case of incongruent TTE findings regarding AS severity, TEE is recommended to directly perform planimetry of the geometric valve orifice area (GOA) and to analyze the morphology of the aortic valve and aortic root [124]. The GOA should be measured at mid-systole with proper alignment of the representative sectional plane, preferably by zoomed 3D data sets. The GOA is limited due to its dependency on forward LVSV. A low LVSV can lead to incomplete AV opening leading to underestimation of GOA, which will result in overestimation of AS severity. The underestimation of GOA in AS patients with AF is independent of the use of a 2D or 3D TEE. The functional hemodynamic effects of AS in AF are characterized by the mean EOA and/or GOA over several cardiac cycles rather than the maximum possible EOA and/or GOA at the maximum possible forward LVSV. Mean values are obviously lower than by the maximum values of VTI_AV_, PG_mean_, and EOA calculated according to the continuity equation in AF [125].

Recent data criticize the approach of averaging 5–10 cycles by arguing that AF is a state of low flow by itself [126], especially in the presence of further AF related morphological and/or functional disorders like LA enlargement and consecutive MR [127].Under these circumstances the single-highest velocity of the transvalvular gradient would be the most accurate approach to diagnose severe AS. This corresponds to the finding, that AV calcium scores obtained by computed tomography (CT) scans in AF patients undergoing TAVI are discordant to echocardiographic PG_mean_ [128]. This hypothesis is supported by a recent study investigating PG_mean_ and AV weight of excised valves in patients with SR or AF [129]. In the case of borderline severe AS flow-independent measurements like 3D TEE evaluation or CT aortic valve calcium scores can be performed to avoid underestimation of AS severity which could lead to deterioration of the patient’s prognosis.

While the extent of LA contractile function has been examined in detail in MS patients, and has been found to be prognostically relevant for AF occurrence and heart failure [130–133], methodological details regarding the determination of MV opening areas (MVOA) or MV regurgitation orifice areas (MVROA) in AF are not stated. According to current guidelines, the MVOA should be determined in AF patients using MVA planimetry in the parasternal short axis by 2D echocardiography or by postprocessing using 3D data sets, as well as indirectly by applying the PHT method or the continuity equation [134–137]. Since heart rate is one important determinant of LV filling with its consecutive effects on contractile LV function and CO, the negative effect of AF on LV filling due to the loss of LA contribution is exacerbated by the presence of MS, which represents a structural component of LV filling obstruction. The loss of atrial contribution in AF patients results in a monophasic inflow, which per se corresponds to restrictive LV filling dynamics. Although the transmitral VTI generally decreases when switching from SR to AF, the forward LVSV will also decrease significantly due to suddenly reduced LV filling with the consequence of backward HF. However, even in permanent AF, the higher the heart rate and the shorter the diastolic interval, the more impaired the LV filling.

Like LV systolic function, MVOA also varies depending on the cycle length in AF. In extremely short RR intervals insufficient driving forces of the LV inflow are present. Under these circumstances, the mitral valve can only open partially or even not at all, so that a smaller MVOA is mostly measured by planimetry than the maximum possible MVOA. This means that planimetry of the MVOA in AF patients with MS is very error-prone, especially if data sets need to be acquired with high frame or volume rates in case of rapid valve motion to be able to determine the maximum MVOA within each RR interval. Therefore, it is obvious that in AF patients with MS the functional relevance of MS can only be assessed by averaging MVA, PHT, and transmitral VTI rather than by the maximum respective values after the index beat method in the presence of sufficient LV filling after a preceding long RR interval. A decrease in MVOA is also accompanied by a decrease in the transmitral PG_mean_ with a reduction of LV filling and a consecutive reduction of LVSV and CO. Whereas MS severity assessed by MVOA planimetry is more likely to lead to a proper assessment of MS severity in AF patients, a decrease of transmitral VTI with AF onset or increasing heart rate during AF are also important due to their consecutive negative effects on LVSV and CO. Therefore, when analyzing the cardiac functional status in AF patients with MS, quantitative LVSV and CO assessment must be performed with respect to rhythm and heart rate.

AF is also not uncommon in AR patients. In large prospective and observational studies the frequency of AF in patients with moderate and severe AR ranges from 14 to 23% [138, 139] In AF patients, significant fluctuations in LV volumes and LVEF can be observed in different cardiac cycles—especially if there is an alternation between pronounced brady—and tachycardic episodes. Reliable quantitative measurements of LVEDV, LVESV, and total and effective LVSV (LVSV_tot_, LVSV_eff_) by 2D and 3D echocardiography determined by averaging measurements in a minimum of five consecutive beats [20] or using the index-beat approach [140] are suitable to estimate regurgitant volume in AF patients with AR. However, AR severity itself significantly depends on heart rate and duration of diastole. Early studies have shown a decrease in regurgitant volume per heart beat with increasing heart rate [141]. Therefore in acute AR, the target heart rate should be between 80 and 100 bpm and bradycardia should be avoided [142]. Little is known about the reliability of semi-quantitative parameters and proximal isovelocity area in AF patients. The current ESC/EACVI position paper on native valvular regurgitation does not address this specific challenge in the quantification of valvular defects [143]. However, AR severity can be better assessed after normalization of the heart rate whenever feasible in AF patients.

Some AF patients show evidence of MR despite structurally normal mitral leaflets and normal LV systolic function and size. This increasingly recognized entity of atrial functional MR is related to LA and mitral annular dilatation with insufficient leaflet coaptation as well as impaired mitral annular dynamics. SR restoration allows gradual recovery of mitral annular dynamics in addition to reverse LA remodeling with decreases in LA and mitral annular dimensions [144]. Just as any significant valvular lesion is associated with AF, so is MR, causing both LA enlargement and increase in LA pressure. With current understanding of complex interaction between the diseases, it seems helpful to first look at primary MR (PMR) to apprehend a first line of cause and effect. Patients with severe PMR and SR at the time of diagnosis will experience an increasing incidence of AF of approximately 5% per year. Aside age as an obvious driver for AF, increasing LA dilatation as a measurement of volume overload in MR patients proved to be an independent AF risk factor [145]. The knowledge on secondary MR (SMR) has been expanded by differentiating the “classical” ventricular SMR (vSMR), caused by dilatative cardiomyopathy of any origin, from the atrial SMR (aSMR) associated with long standing AF [146–148]. The increase in dilation of the mitral annulus found in both subtypes of SMR fuels a vicious circle, further increasing MR, increasing LA pressure and LA volume overload and AF occurrence. It is therefore of most importance to understand the underlying pathology when evaluating a patient with AF also suffering from MR. The first logical step would be to identify a MR ≥ moderate to decide if an accurate analysis is necessary. Explicitly, the MR jet area is only recommended to detect MR of any severity, but not for grading of MR severity especially in AF patients with varying beat-to beat jet areas. Second, if the MR seems ≥ moderate, then the underlying pathology needs to be identified. The accurate assessment of LA, as well as LV volumes and function, is again subject to the general problem of the variability of all results at different heart rates in AF patients. Third, grading of MR severity in moderate vs. severe MR can be achieved by integrating the measurements of multiple quantitative and semi-quantitative variables. Nevertheless, all echocardiographic parameters formally need to be averaged over several heart beats or need to be analyzed within comparable index beats. Imbalance of closing and opening forces, either through mitral annular dilation, or tethering of the mitral leaflets due to LV dilation, will cause SMR. This causes a crescent shaped effective regurgitant orifice area (EROA) since the complete mitral valvular apparatus is affected, as opposed to the localized defect in PMR. It becomes apparent that there is a need for a reliable individual hemodynamic assessment of each AF patient. The calculation of the regurgitant fraction (RF) should theoretically meet this requirement, provided all variables are considered. A relatively easy method to estimate RF in isolated MR is by considering LVSV_eff_ in relation to the regurgitant volume, as assessed by 2D PISA (proximal isovelocity surface area). A RF ≥ 50% indicates severe MR. The LVSV_tot_, as measured by planimetry or volumetry, may serve as an alternative substituting LVSV_eff_ and regurgitant volume. Finally, all findings must be weighed and scrutinized for plausibility, taking into account clinical signs and symptoms of the patients to avoid miscalculation of MR [149]. In SMR, the MR severity in AF is largely determined by the AF-related impairment of LV function. In contrast to AR the regurgitant volume in SMR patients per cardiac cycle remains almost the same in AF episodes. Thus, if LVSV_tot_ decreases in SMR patients due to ongoing AF, LVSV_eff_ significantly decreases in the presence of nearly constant regurgitant volumes—especially in tachyarrhythmia. This mechanism induces and maintains progressive cardiac failure in SMR patients.

## Summary

This expert proposal focusses on the lack in current recommendations addressing the challenges of echocardiography in AF patients. Imaging by echocardiography usually represents the first diagnostic step to assess atrial, ventricular, and valvular function as well as to clarify the cause of AF in patients with paroxysmal, permanent, and persistent AF and in patients with suspected history of AF.

## Electronic supplementary material

Below is the link to the electronic supplementary material.

Supplementary file1 (PDF 1741 KB)

Supplementary file2 (DOCX 29 KB)

## Appendix: conference discussion

Prof. Dr. Andreas Hagendorff (Leipzig): One major topic at the “Deutscher Echokardiographie Kongress 2023” in Leipzig was echocardiography in AF patient. Therefore, the present expert proposal deals with the topic of analyzing the pathophysiology of cardiac diseases in atrial fibrillation using echocardiography. ***To start the conference discussion—do you believe that the importance of cardiac imaging is adequately presented in the current guidelines for the diagnosis and treatment of atrial fibrillation?***

Dietrich Pfeiffer (Berlin): In my opinion, the topic of this expert proposal focuses on an obvious gap in the current recommendations about diagnosis and treatment of AF. Therefore, this manuscript will certainly attract great interest. ***What are the most important tasks of echocardiography in the context of atrial fibrillation?***

Roland Brandt (Bad Nauheim): The most important tasks of echocardiography in AF patients are the possible clarification of the etiology of AF and the individual assessment of cardiac function. The detection of thrombi we will discuss later. ***To determine LV systolic function in AF patients, which echocardiographic parameter seems most important to you and for what reason?***

Ali Hamadanchi (Jena): In clinical routine, the assessment of LVEF is still the most frequently used and therefore most important parameter for assessing systolic function. However, subclinical changes can certainly be better detected by GLS. ***Regarding the changing RR cycles, can parameters such as LV volume, LVEF and LV strain be averaged over several cardiac cycles or determined using the index-beat method? What speaks for which procedure under which conditions?***

Nicolas Merke (Berlin): The echocardiographic parameters in AF patients can be determined by time-consuming averaging over several cardiac cycles or by the index beat approach. In principle, however, when interpreting the data, it must always be considered that in the case of averaging over several cardiac cycles, the presumably real functional status is depicted, and with the index beat approach, the best possible functional cardiac state is depicted. ***The similar question to you. Which echocardiographic parameter do you think is most important to determine LV diastolic function in AF patients, and for what reason?***

Thomas Groscheck (Magdeburg): Important parameters of diastolic function are the indexed LA volume and E/E´, for which, however, there are different cut-off values ​​than for patients in SR. In addition, the systolic pulmonary pressure should be measured as a minimum requirement to clarify effects of the respective cardiovascular diseases with AF on pulmonary circulation. ***What is your practical recommendation general for interpreting echocardiographic results of systolic and diastolic function in the presence of high heart rate variability during AF?***

Sebastian Ewen (Homburg/Saar): The presence of high heart rate variability AF makes it difficult to achieve reproducible echocardiographic values. Therefore, echocardiographic assessment should be performed after rate control – if possible. Averaging of volumetric and functional parameter measurements should be performed in high heart rate variability to characterize the individual functional state of the AF patient. It is also advisable to use multiplanar and multidimensional echocardiographic techniques to increase the accuracy of cardiac volumetric and functional echocardiographic measurements. **Currently, the LA size should be analyzed at least biplane or by multidimensional echocardiography. Do you think that the current values ​​for LAVI**_**max**_** are correct with respect to modern technologies, and why does LAVI**_**min**_** appear to be potentially the more interesting parameter than LAVI**_**max**_** for you?**

Henrik ten Freyhaus Köln): Clearly, LAVI_max_ has limitations, for example due to the known increase of LA volume with age. However, it still represents the most robust parameter for LA size. Due to a clear association with outcome in AF, LAVI_min_ may be pathophysiologically more relevant. Limitations of this parameter include the lower reproducibility and inter-observer variability. This underscores the critical importance of standardization in echocardiography. Using 3D echocardiography, it is likely that normal values of LAVI are higher and need to be redefined. ***What are even today the advantages of conventional analysis of LA function using MAPSE, LA emptying fraction, behavior of the interatrial septum and LA size?***

Elena Romero-Dorta (Berlin): The conventional parameters to evaluate LA function and size are mostly easy to assess and show prognostic value. MAPSE is a high reproducible, feasible and sensitive marker for LV dysfunction. Total LAEmF can be calculated after measuring the LA maximum and minimum volume using 2- or 3-D TTE. Therefore, LAEmF should be measured either way in a standard examination, at least LA maximum volume. LAEmF also reflects the reservoir function and has shown predictive value in terms of AF recurrence after cardioversion. The directional movement of the interatrial septum and its curvature, a gaze observation, is an easy and fast way to assess the pressure relation between LA and RA indirectly. ***The relatively unknown parameter of the PA-TDI interval is rarely used. But why does it seem particularly important in patients with paroxysmal AF or in patients with yet unrecognized AF?***

Stephan Beckmann (Berlin): PA-TDI as a parameter of the time interval between electrical LA activation and mechanical LA contraction is easy to measure and seems to be useful to predict AF in patient with embolic stroke of unknown source. In addition, PA-TDI can provide risk-stratified information. ***In the literature LA strain is sometimes maliciously claimed as only caused by the LV deformation alone, since it shows opposite deformation curves. Why do you believe in the independent importance of the parameter LA strain?***

Fabian Knebel (Berlin): Certainly, the atrial deformation interacts with the ventricular deformation. But the comparison of patients, e.g. with hypertensive heart disease versus storage diseases, with the same LV GLS and different impairment of the LA reservoir strain, proves the independent importance of the LA strain.

Prof. Dr. Andreas Hagendorff (Leipzig): ***An additional methodological question: Should the LA strain only be measured in the LA areas near the mitral ring or everywhere in the LA contour—including in the regions of the IAS and the confluence of the LAA and the upper pulmonary vein?***

Fabian Knebel (Berlin): Due to the methodological limitations described, I personally prefer to only evaluate the LA strain within the areas near the mitral ring—as shown in our Fig. 2. However, this methodological approach is not considered in the current recommendations. ***Valvular heart diseases lead to valve defects that significantly affect hemodynamics. If one considers the interaction of LV filling and LV ejection as a function of atrial contribution and heart rate, what are the general implications for the echocardiographic analysis of our patients—especially when changing from SR to AF?***

*Thomas Binder (Wien):* Especially in the case of valvular defects, attention must also be paid to effective CO, if the ventricular filling is impaired. Therefore, the LVSV_eff_ should always be estimated from the LVOT- and/or RVOT- forward stroke volume under AF conditions. Gradients across the valves can underestimate and overestimate the severity of valve defects depending on heart rate. ***When determining the AS severity in AF patients, there is the problem of incomplete AV opening in rapid cardiac cycles with low LV ejection and consecutive limited LV filling. This would correspond to pseudo-AS. In these cases, do you consider AS with AVA values ​​ > 1cm***^***2***^*** at maximum AV opening to be moderate, even if the mean AVA in these AF patients is obviously < 1.0cm***^***2***^***?***

Sebastian Kruck (Ludwigsburg): In patients with tachyarrhythmia planimetric aortic valve opening as well as pressure gradients underestimate the GOA due to the reduced forward stroke volume. Therefore, I would not rely on a single assessment of GOA in tachyarrhythmia patients with AS. In this case, an additional TTE or TEE examination is required, if possible, when the patient has a normal heart rate, or after cardioversion in SR. Depending on the clinical situation, the AS severity must then be interpreted depending on the maintenance of a stable SR or at normofrequent AF. ***Rheumatic or degenerative mitral valve stenosis represents a special situation. MS is a structural obstacle that leads to LA dilatation and LA fibrosis as well as massive LA dysfunction even in SR. What practical recommendations do you have for MS severity assessment in AF patients?***

Christoph Sinning (Hamburg): As already indicated in the discussion, in MS there is a close relation of CO on LV filling, which is of course extremely unfavorable with fast heart rates. In rheumatic MS, surprisingly high transmitral gradients can be observed, so that the limits of individual compensation only become apparent when AF occurs. In all these cases of chronic adaptation, an estimation of CO under light exercise, e.g. after squats, can be very helpful in characterizing the clinical relevance of MS. ***In the literature, a decrease of regurgitation volume in AR is described with increasing heart rate. Can you explain the pathophysiological reason for this, and list any possible disadvantages of the higher heart rate in the presence of AR in AF patients?***

Jan Knierim (Berlin): LV filling in AR occurs physiologically transmitrally and pathologically retrogradely via the aortic valve during diastole. With longer diastole, the transmitral inflow is increasingly hindered by the retrograde inflow via the aortic valve, so that in the case of relevant AR, a pressure equalization between the left atrium and the ascending aorta can occur. This is observed with a shorter deceleration time than the diastole duration, when the cw regurgitation signal reaches the zero line prior to ejection period. On the other hand, in the presence of very fast heart rate failure of aortic valve closure can occur with the effect of ongoing regurgitation over several RR intervals due to inadequate LV filling. ***Can you explain why there are functional differences in the regurgitation volume-heart rate relationship between AR and MR patients?***

Tobias Ruf (Mainz): The fundamental difference in the pathophysiological effects of AR and MR is that the aortic valve is within the high-pressure system, whereas the mitral valve is the border between the low and high-pressure system of the circulation. Because of this difference, a hemodynamically comparable MR is usually more clinically relevant than an AR with comparable regurgitation fraction. The interpretation of the echocardiographic findings becomes difficult in the presence of both, MR and AR, since MR is aggravated both, by the eccentric LV dilatation with possible mitral annulus dilatation and by the increase in systolic LV pressure induced by AR. ***However, to finalize our discussion what is the most important message of the present expert proposal for assessing LA and LV function in AF patients by echocardiography?***

*Andreas Hagendorff (Leipzig)* *:* *Echocardiography in AF patients is fundamentally challenging due to the difficulties resulting from heart rate variability, especially since the interpretation of the quantitative functional parameters depends on the respective evaluation modalities. Overall, when assessing echocardiographic parameters, attention must also be paid to clinical and hemodynamic plausibility. Thus, the duty of care as well as education and training in echocardiography are the cornerstones of good patient care.*
