# Supplementary figures and images for: Echocardiographic assessment of atrial, ventricular, and valvular function in patients with atrial fibrillation—an expert proposal by the german working group of cardiovascular ultrasound
Source: Clin Res Cardiol. 2024 Aug 26;114(1):4–24. doi: 10.1007/s00392-024-02491-6 (PMC11772422; doi:10.1007/s00392-024-02491-6)

Figure 1 - Supplement:

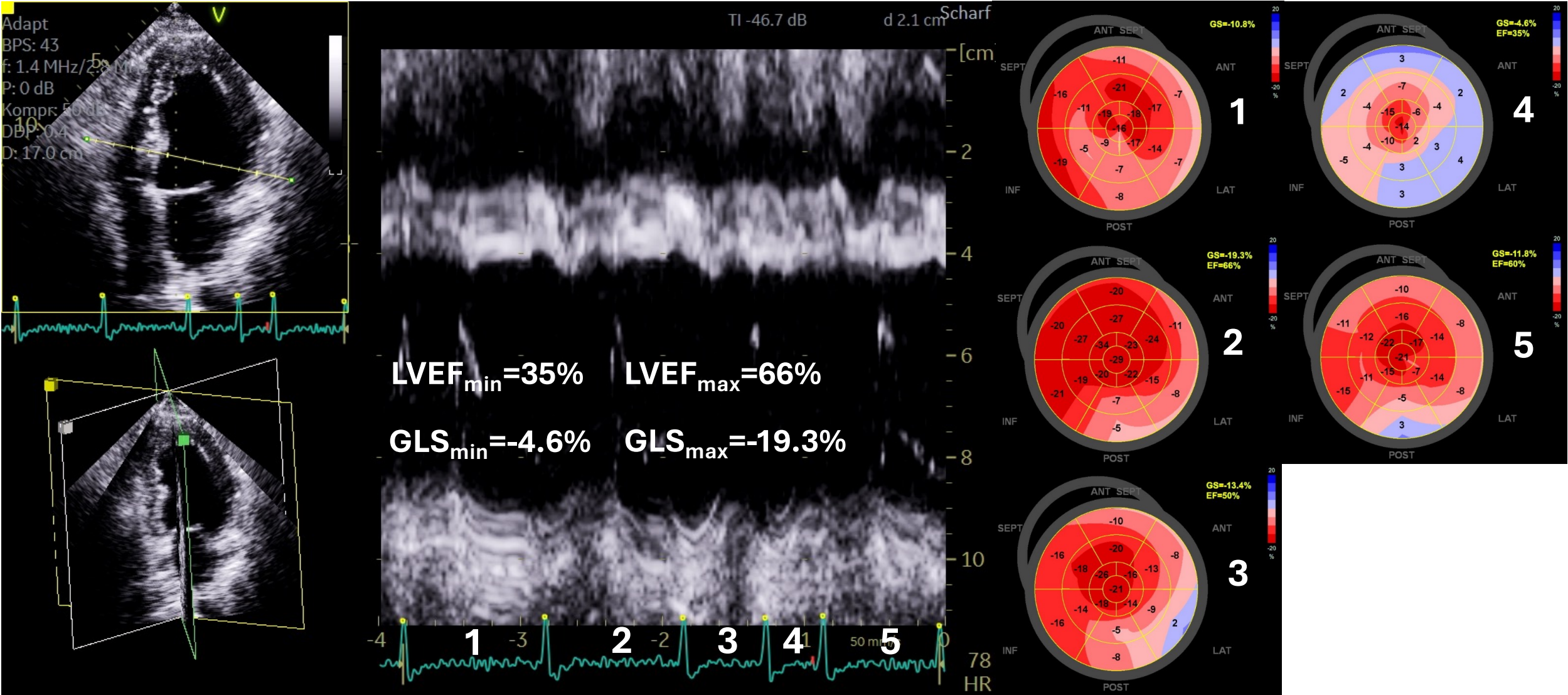

Figure 2 - Supplement:

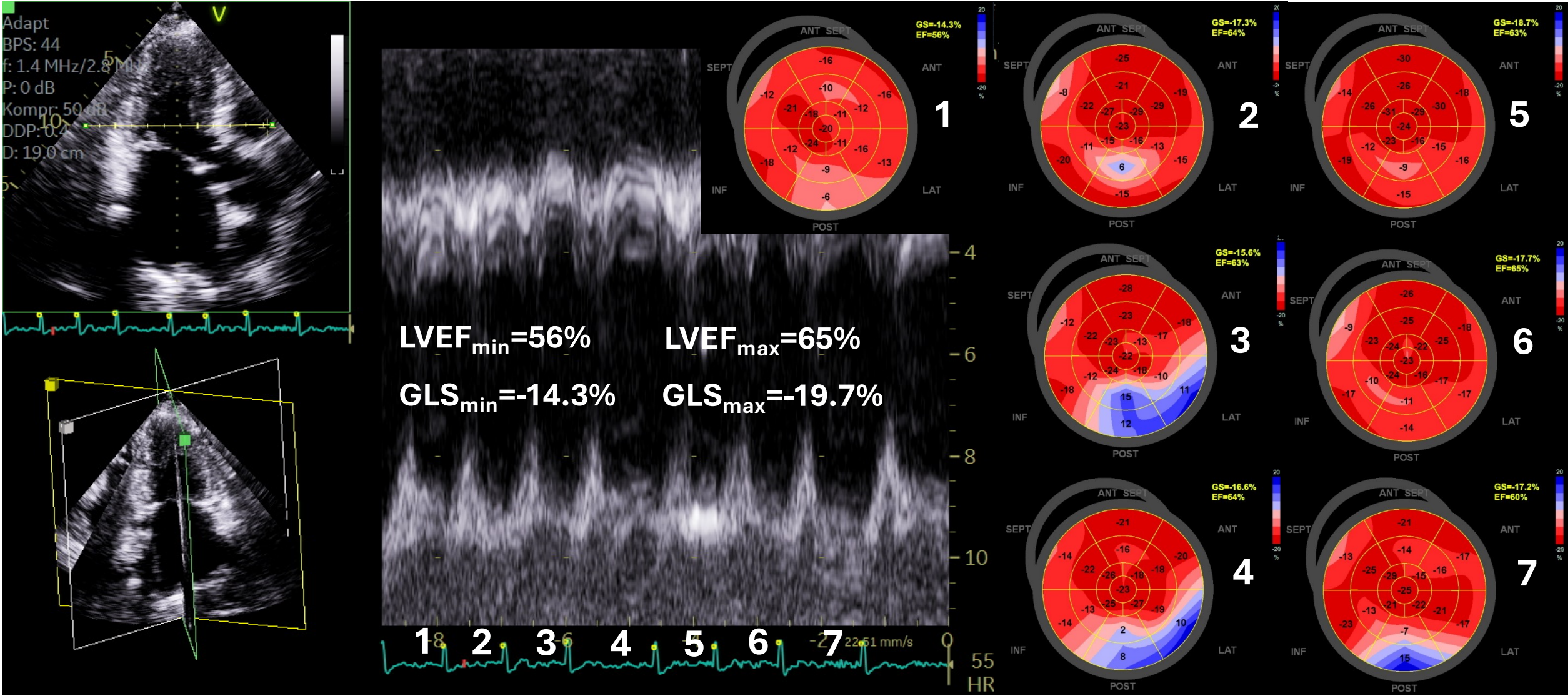

Figure 3 - Supplement:

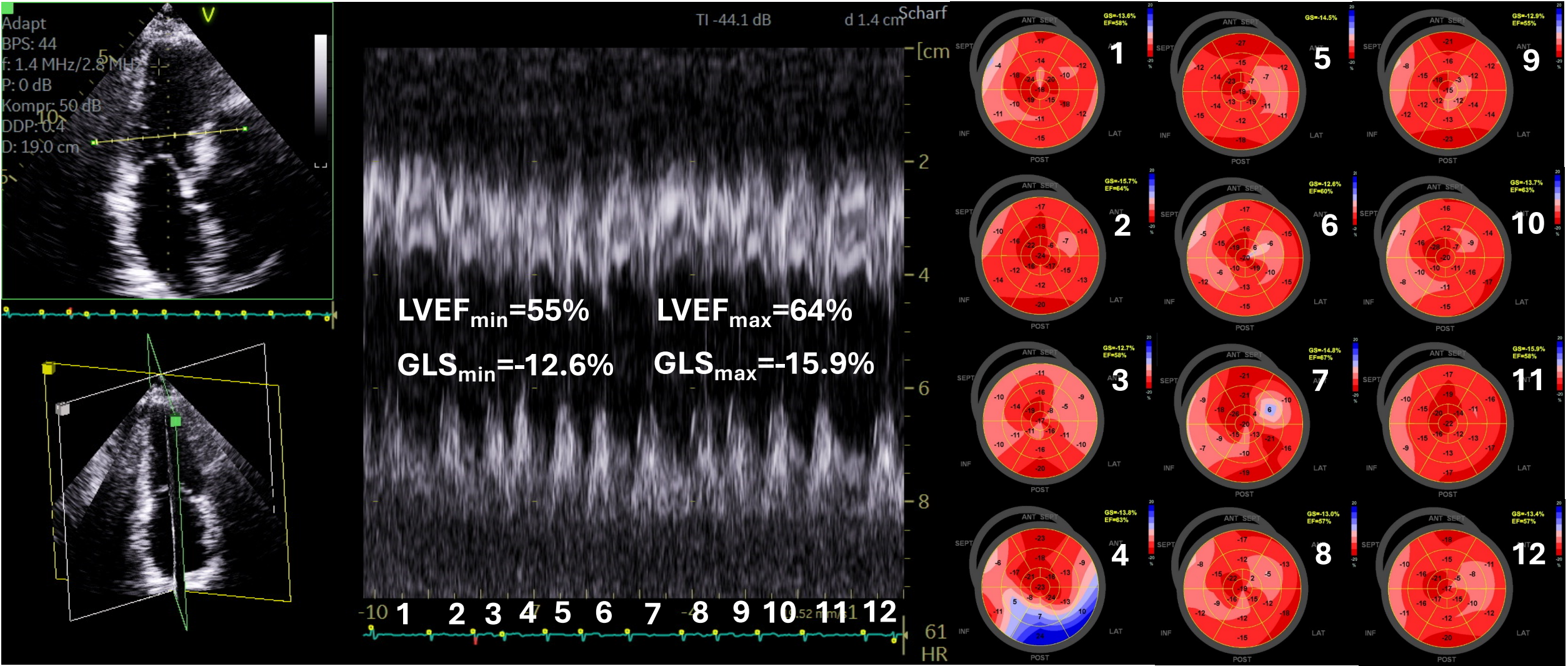

Supplement: Supplementary file 1 — Supplementary file1 (PDF 1741 KB) [file 392_2024_2491_MOESM1_ESM.pdf]
