# Supplementary material for: Echocardiographic assessment of atrial, ventricular, and valvular function in patients with atrial fibrillation—an expert proposal by the german working group of cardiovascular ultrasound
Source: Clin Res Cardiol. 2024 Aug 26;114(1):4–24. doi: 10.1007/s00392-024-02491-6 (PMC11772422; doi:10.1007/s00392-024-02491-6)
Supplement: Supplementary file 2 — Supplementary file2 (DOCX 29 KB) [file 392_2024_2491_MOESM2_ESM.docx]

**Analysis of LA dysfunction during sinus rhythm (SR) in patients with paroxysmal AF or during AF in persistent AF**

In paroxysmal AF, LA function can be assessed during SR using the maximum transmitral blood flow velocity during atrial contraction by pw Doppler (V_max_A) [1]. The V_max_A in the transmitral pw Doppler spectrum reflects the LA-LV-gradient in late diastole and is influenced by the contractile function and the compliance of the left ventricle. A higher A wave indicates a stronger LA pump function. However, the diagnostic value of the V_max_A for assessing LA function is limited due to various influencing factors. The maximum velocity of the A wave increases with age [1–3]. Heart rate, stress dependency and the LV compliance can also influence this parameter. In sinus tachycardia or AV block, the E and A waves may merge so that the E and A waves are indistinguishable.

With decreasing LV relaxation and increasing LV stiffness, the relative importance of LA contraction increases with increasing LVEDP which leads to a decrease of the conduit function. As LVEDP continues to increase, preload reserve can be achieved by LA contraction, resulting in LA behaving predominantly like a conduit. Accordingly, the V_max_A for increases in early stages of DD where relaxation disturbances dominate. When the LVEDP increases in later stages of DD, the V_max_A again decreases [1]. The maximum systolic basal myocardial velocity during atrial contraction (V_max_A´), recorded with tissue pw Doppler is a surrogate for LA contractile function. The velocities are similar for septal and lateral measurements, so that measurements can be made at both locations of the MV annulus. As with the A wave, V_max_A' can also increase with age, which is due to a decrease in LV compliance with age [1, 2]. However, the clinical significance is limited, among other things, by the fact that the tissue Doppler cannot distinguish between active atrial contraction by LA translational movements and passive movement of the MV annulus. The total atrial conduction time (TACT) can be measured by the time interval between the onset of the P-wave and the peak V_max_A' (PA-TDI) [4]. This parameter characterizes the time between electrical LA activation and mechanical LA contraction. An increase in atrial conduction time could be caused by impaired LA function. Normal ranges of PA-TDI are between 100 and 150ms [5]. It has been proven that increased LA conduction time can predict AF recurrence after cardioversion or radiofrequency catheter ablation [6–9].

During SR phasic LA function can be described by the reservoir function in systole, the conduit function in early diastole and the booster pump function in late diastole. Reservoir function is influenced by the LA and LV relaxation, the LA compliance and the motion of the LV base during systole, while booster function is influenced by LA contractility and LV enddiastolic compliance and LV pressure [10]. Increased LVEDP and increased LA afterload can reduce LA reservoir function and LA strain. In this case, LA strain correlates with LVEDP [11], while LA reservoir function shows a linear decrease with higher stages of LV DD [10]. If LVEDP is increased peak LA strain is < 20% (normal value of LA reservoir strain: 39%)[12]. With a cut-off value of < 18% LA reservoir strain is part of the HFpEF algorithm to estimate LV filling pressures [13]. During AF episodes or in persistent/permanent AF A-wave and A´-wave are not present and therefore cannot be assessed. Due to this fact, the LV filling shows a monophasic flow profile, which formally corresponds to restrictive LV filling states.

References:

1. Nagueh SF, Smiseth OA, Appleton CP, et al (2016) Recommendations for the Evaluation of Left Ventricular Diastolic Function by Echocardiography: An Update from the American Society of Echocardiography and the European Association of Cardiovascular Imaging. Journal of the American Society of Echocardiography 29:277–314. https://doi.org/10.1016/j.echo.2016.01.011

2. Nagueh SF (2020) Left Ventricular Diastolic Function: Understanding Pathophysiology, Diagnosis, and Prognosis With Echocardiography. JACC Cardiovasc Imaging 13:228–244. https://doi.org/10.1016/j.jcmg.2018.10.038

3. Nagueh SF (2020) Diastology: 2020-A practical guide. Echocardiography 37:1919–1925. https://doi.org/10.1111/echo.14742

4. Merckx KL, De Vos CB, Palmans A, et al (2005) Atrial activation time determined by transthoracic Doppler tissue imaging can be used as an estimate of the total duration of atrial electrical activation. J Am Soc Echocardiogr 18:940–944. https://doi.org/10.1016/j.echo.2005.03.022

5. Müller P, Weijs B, Bemelmans NMAA, et al (2021) Echocardiography-derived total atrial conduction time (PA-TDI duration): risk stratification and guidance in atrial fibrillation management. Clin Res Cardiol 110:1734–1742. https://doi.org/10.1007/s00392-021-01917-9

6. den Uijl DW, Gawrysiak M, Tops LF, et al (2011) Prognostic value of total atrial conduction time estimated with tissue Doppler imaging to predict the recurrence of atrial fibrillation after radiofrequency catheter ablation. Europace 13:1533–1540. https://doi.org/10.1093/europace/eur186

7. De Vos CB, Weijs B, Crijns HJGM, et al (2009) Atrial tissue Doppler imaging for prediction of new-onset atrial fibrillation. Heart 95:835–840. https://doi.org/10.1136/hrt.2008.148528

8. Antoni ML, Bertini M, Atary JZ, et al (2010) Predictive value of total atrial conduction time estimated with tissue Doppler imaging for the development of new-onset atrial fibrillation after acute myocardial infarction. Am J Cardiol 106:198–203. https://doi.org/10.1016/j.amjcard.2010.02.030

9. Buck T, Breithardt O-A, Faber L, et al (2009) Manual zur Indikation und Durchführung der Echokardiographie. Clin Res Cardiol Suppl 4:3–51. https://doi.org/10.1007/s11789-009-0051-6

10. Thomas L, Marwick TH, Popescu BA, et al (2019) Left Atrial Structure and Function, and Left Ventricular Diastolic Dysfunction: JACC State-of-the-Art Review. J Am Coll Cardiol 73:1961–1977. https://doi.org/10.1016/j.jacc.2019.01.059

11. Gan GCH, Ferkh A, Boyd A, Thomas L (2018) Left atrial function: evaluation by strain analysis. Cardiovasc Diagn Ther 8:29–46. https://doi.org/10.21037/cdt.2017.06.08

12. Singh A, Medvedofsky D, Mediratta A, et al (2019) Peak left atrial strain as a single measure for the non-invasive assessment of left ventricular filling pressures. Int J Cardiovasc Imaging 35:23–32. https://doi.org/10.1007/s10554-018-1425-y

13. Smiseth OA, Morris DA, Cardim N, et al (2022) Multimodality imaging in patients with heart failure and preserved ejection fraction: an expert consensus document of the European Association of Cardiovascular Imaging. Eur Heart J Cardiovasc Imaging 23:e34–e61. https://doi.org/10.1093/ehjci/jeab154
